# A Surface Protein From *Lactobacillus plantarum* Increases the Adhesion of *Lactobacillus* Strains to Human Epithelial Cells

**DOI:** 10.3389/fmicb.2018.02858

**Published:** 2018-11-22

**Authors:** Guangqiang Wang, Minghui Zhang, Jianxin Zhao, Yongjun Xia, Phoency F.-H. Lai, Lianzhong Ai

**Affiliations:** ^1^Shanghai Engineering Research Center of Food Microbiology, School of Medical Instrument and Food Engineering, University of Shanghai for Science and Technology, Shanghai, China; ^2^State Key Laboratory of Food Science and Technology, School of Food Science and Technology, Jiangnan University, Wuxi, China

**Keywords:** *Lactobacillus*, surface protein, GAPDH, adhesion, HT-29

## Abstract

Adhesion to epithelial cells is considered important for *Lactobacillus* to exert probiotic effects. In this study, we found that trypsin treatment decreased the adhesion ability of *Lactobacillus plantarum* AR326 and AR269, which exhibit good adhesion ability, and surface proteins extracts increased the adhesion of the strains with poor adhesion ability. By SDS–polyacrylamide gel electrophoresis and mass spectrometry analysis, the main component of the surface proteins was detected and identified as a protein of approximately 37 kDa. It was 100% homologous with glyceraldehyde-3-phosphate dehydrogenase (GAPDH) from *L. plantarum* WCFS1. The adhesion of AR326 and AR269 was decreased significantly by blocking with the anti-GAPDH antibody, and GAPDH restored the adhesion of AR326 and AR269 treated with trypsin. In addition, purified GAPDH significantly increased the adhesion of the strains with poor adhesion ability. These results indicated that GAPDH mediates the adhesion of these highly adhesive lactobacilli to epithelial cells and can be used to improve the adhesion ability of probiotics or other bacteria of interest.

## Introduction

*Lactobacillus* is the most common probiotics which can confer beneficial effects on their hosts when consumed in adequate amounts. These beneficial benefits may include preventing diarrhea (Staudacher, 2015; Sinclair et al., 2016; Kim et al., 2018), modulating the intestinal community (Marteau et al., 2002; Xie et al., 2016), improving lactose tolerance (Mustapha et al., 1997; Staudacher, 2015; Oak and Jha, 2018), reducing the serum cholesterol level (Choi et al., 2015; Kobyliak et al., 2016), suppressing pathogens (Kim et al., 2014; Tuo et al., 2018), and boosting the immune system (Dimitrijevic et al., 2014; Kemgang et al., 2014). To maximize these functions, *Lactobacillus* strains must be able to adhere to and colonize the gastro-intestinal tract (Ljungh and Wadstrom, 2006; Tuo et al., 2018). Therefore, one important criterion in selecting probiotic bacteria is their ability to adhere to epithelial cells of the intestinal tract.

Adhesion is a specific interaction between the surface components of lactobacilli and intestinal surfaces. The adhesion abilities of lactobacilli have been linked with various different surface components including (lipo) teichoic acids, polysaccharides, and proteins (Deepika and Charalampopoulos, 2010; Yadav et al., 2017). Surface proteins have been reported to play an important role in the adherence of lactobacilli to the host. After the removal of surface proteins from lactobacilli, such as by trypsin treatment, the adhesive ability of lactobacilli decreases (Deepika and Charalampopoulos, 2010; Glenting et al., 2013; Zhang et al., 2013). Many diverse groups of these surface proteins have been identified. Proteins containing a C-terminal sortase recognition motif (LPXTG) from different *Lactobacillus* strains have been identified and shown to bind to mucus and epithelial cells (Pretzer et al., 2005; Jensen et al., 2014). Other adhesins, including the collagen-binding protein CnBP, mucin-binding protein MapA, and the fibronectin-binding proteins FbpA, have been shown to adhere to the extracellular matrix proteins such as collagen, laminin, and fibronectin (Buck et al., 2005; Miyoshi et al., 2006).

Recently, many unforeseen proteins on the outer cell surface of different *Lactobacillus* strains have been identified. These proteins are mainly conserved cytoplasmic proteins, such as elongation factor Tu, the chaperonin protein complex GroEL, and glyceraldehyde-3-phosphate dehydrogenase (GAPDH) (Dhanani and Bagchi, 2013; Glenting et al., 2013; Zhu et al., 2016). The most identified proteins can bind to the extracellular matrix components, such as collagen, laminin, and fibronectin. It is generally thought that by binding to these host components, these proteins help lactobacilli adhere to host cells (Deepika and Charalampopoulos, 2010; Dhanani and Bagchi, 2013; Wang et al., 2014). Because the canonical functions of these proteins inside the cell are in essential cellular processes, such as glycolysis, protein synthesis, and chaperones, the creation of a knockout mutant is impossible. Thus, few studies have provided direct experimental evidence for the role of these cell surface proteins in the adhesion of *Lactobacillus* to the host cells. In addition, although there have been many studies of the effect of the surface proteins on the adhesion of their originating strains (Von Ossowski et al., 2011; Yadav et al., 2017), few studies have been conducted on their effect on other strains or on how to improve adhesion, especially of strains with low adhesion ability (Avall-Jaaskelainen et al., 2003; Zhang B. et al., 2015). In this study, we investigated the key surface protein mediating the adhesion of the strains with high adhesive ability to intestinal epithelial HT-29 cells and explored the effect of this surface protein on the adhesion ability of other strains with and without further treatment.

## Materials and Methods

### Bacterial Strains, Cells, and Culture Conditions

Isolated *Lactobacillus plantarum* AR326, AR269, AR171 and *Lactobacillus paracasei* AR187 were stored in the Shanghai Engineering Research Center of Food Microbiology (Shanghai, China). They were previously screened and identified using the 16S rDNA and showed over 96% sequence similarity with *L. plantarum* WCFS1 and *Lactobacillus paracasei* M0116, separately. The 16S rDNA between them showed a slight difference (Zhang et al., 2017). All *Lactobacillus* strains were anaerobically cultured in deMan, Rogosa, and Sharpe (MRS) broth (Difco) at 37°C and collected at logarithmic growth phase for later use.

*Escherichia coli* BL21 (DE3) [Takara Bio (Dalian) Co., Ltd., Dalian, China] used for recombinant expression was grown at 37°C in LB broth supplemented with 50 μg/mL of kanamycin when appropriate. Human adenocarcinoma cell line HT-29 was purchased from the Cell Resources Center of Shanghai Institutes for Biological Sciences (Shanghai, China). HT-29 cells were cultivated in 1640 medium (Gibco,^TM^ Thermo Fisher Scientific, Grand Island, NY, United States) containing 10% fetal bovine serum at 37°C in 5% CO_2_.

### Detection of Lactobacilli Surface Hydrophobicity and Auto-Aggregation

Cell surface hydrophobicity was determined as the affinity to hydrocarbons, using the xylene extraction method following Collado et al. (2008). Auto-aggregation assays were carried out as described previously (Collado et al., 2008).

### *Lactobacillus* Adhesion Experiment on HT-29 Cells

The concentration of the *Lactobacillus* suspension was adjusted to 10^8^ cfu/mL. When needed, the *Lactobacillus* was firstly treated with LiCl, or trypsin, then determined the adhesion as following. HT-29 cells were cultured on 12-well plates. When they covered 80–90% of the plates, they were rinsed twice with PBS and 1 mL of *Lactobacillus* suspension and 1 mL of 1640 culture medium was added to each well. The plates were placed in a cell incubator at 37°C for 2 h, then rinsing with PBS three times, methanol fixation (1 mL) for 20 min, PBS rinsing twice, gram staining, and microscopic observation. Twenty views were randomly chosen for imaging. The number of *Lactobacilli* adhering to HT-29 cells was calculated on the basis of 100 cells. The experiment was repeated in triplicate.

### Treatment of *Lactobacillus* Strains With Lithium Chloride and Trypsin

To isolate surface proteins, *Lactobacillus* strains were cultivated anaerobically in MRS broth (Difco) at 37°C for 18 h (at logarithmic growth phase). Total surface protein extracts using 5 M LiCl (BBI, China) were prepared following the method of Lortal et al. (1992). Acquired surface proteins were dialyzed for 36 h at 4°C against distilled water using 2 kDa cut-off membranes (Shanghai Yuanye Bio. Co., Shanghai, China), then used for SDS–polyacrylamide gel electrophoresis (PAGE) analysis and added to the different strains for later testing. The pellets of the lactobacilli treated with LiCl were adjusted to 10^8^ cfu/mL with 0.01 mol/L PBS for the adhesion experiment. Protein concentrations were determined by using the Bradford assay (Olson and Markwell, 2007).

The *Lactobacillus* strains were treated with trypsin according to Zhu et al. (2016). The pellets of the lactobacilli treated with trypsin were collected and adjusted to 10^8^ cfu/mL with 0.01 mol/L of PBS for the adhesion experiments.

### SDS–PAGE Analysis and Protein Identification

Total surface protein extracts were separated by 12% Tris–HCl SDS–PAGE (Ventura et al., 2002). The gels were stained with Coomassie brilliant blue (Bio-Rad, Hercules, CA, United States) to visualize separated proteins and bands were analyzed using the Gel Doc XR imaging system (Bio-Rad, Hercules, CA, United States). The 37 kDa band was excised using a sterile razor blade and used for mass spectrometry analysis using a MALDI-TOF-TOF instrument (4800 proteomics analyzer; Applied Bio-systems, United States) by Sangon Biotech (Shanghai) Co., Ltd. (Shanghai, China).

### Cloning, Expression, and Purification of *GAPDH*

The *GAPDH* gene was amplified from *L. plantarum* AR326 chromosomal DNA using primers of GapAF (5’-GGGAATTCCATATGTCTGTAAAAATTGGTATTAATG-3’, *Nde*I site underlined) and GapAR (5’-TACCGCTCGAGAGTGGCGAACTTCAAT-3’, *Xho*I site underlined). The amplified PCR product was digested with the endonucleases *Nde*I and *Xho*I and ligated into a pET-28a expression vector (Novagen) previously digested with the same restriction enzymes, resulting in pET24a-GAPDH. For inducible expression, pET24a-GAPDH was transformed into *E. coli* BL21 (DE3). After inducing the log-phase cells cultured at 37°C with 0.5 mM of isopropyl-β-D-thiogalactoside (IPTG, BBI, China) for 2 h, the pellets were harvested by centrifugation and resuspended in ice-cold PBS. The purified protein was obtained using a His-Tagged Protein Purification Kit (CW0894, CWBIO, China). The purity of the sample was verified with SDS–PAGE. Protein concentration was determined by using the Bradford assay (Olson and Markwell, 2007).

The purified GAPDH protein was sent to AbMax Co., Ltd. (Beijing, China) for the generation of a rabbit polyclonal antibody.

### Assay of Adhesion Ability of GAPDH and Anti-GAPDH Antibody Treated *Lactobacillus* Strains to HT-29 Cells

The *Lactobacillus* pellets (if need, they were treated with trypsin) were washed three times with PBS and adjusted to 10^8^ cfu/mL. They were then incubated with the purified GAPDH or anti-GAPDH antibody for 30 min at room temperature. The treated lactobacilli were harvested and washed with PBS and suspended in 1640 medium. Assays to determine the adherence of the lactobacilli to the HT-29 cells after treatment were conducted as described above for untreated lactobacilli.

### Statistical Analysis

Statistical package for social sciences (SPSS) software version 22.0 (SPSS Inc., Chicago, IL, United States) was used to perform statistical analysis. The significance for differences among data were assessed using ANOVA program with the level of significance at *P* < 0.05.

## Results

### Surface Proteins Extracts Increase the Adhesion of Different Strains to HT-29 Cells

**FIGURE 1:**
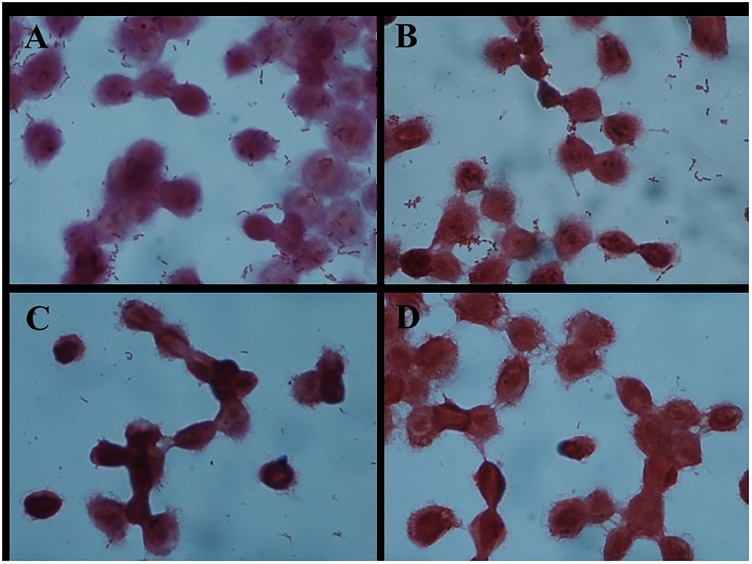
Adhesion of different *Lactobacillus* strains to HT-29 cells as observed by Gram staining under a light microscope (magnification 1000×). **(A)** *L. plantarum* AR326. **(B)** *L. plantarum* AR269. **(C)** *L. paracasei* AR187. **(D)** *L. plantarum* AR171.

To assess the characteristics of the strains, the auto-aggregating, hydrophobicity, and adhering abilities of *L. plantarum* AR326, AR269, AR171 and *L. paracasei* AR187 were studied. Although the difference in the16S rDNA of theses strains was slight, significant differences in these properties between strains were observed (Table 1). The lowest hydrophobicity was observed for AR326 (11.70%), whereas AR326 showed the maximum auto-aggregation rate (45.53%) and best adhesion ability (411 bacterial counts/100 cells, Figure 1). AR171 exhibited the greatest hydrophobicity, but we did not detect its adhesion to HT-29 cells (Figure 1). AR187 had a similar auto-aggregation rate to AR326, but higher hydrophobicity and lower adhesion than AR326. AR269 had similar adhesion ability to AR326, but the hydrophobicity and auto-aggregation rates were quite different. Generally, these properties were strain specific. The auto-aggregation and hydrophobicity of the tested strains had no statistically significant correlations to each other and represented different probiotic effects. This corresponds with the results of previous studies (Vlková et al., 2008; Tuo et al., 2013).

**Table 1:** Surface properties and adhesion abilities of four *Lactobacillus* strains.

Strain	Hydrophobicity (%)	Auto-aggregation rate (%)	Adhesion ability (bacterial counts/100 HT-29 cells)
*L. plantarum* AR326	11.70 ± 6.77^a1^	45.53 ± 1.18^a^	411 ± 12^a^
*L. plantarum* AR269	73.93 ± 7.07^b^	21.01 ± 2.20^b^	394 ± 21^b^
*L. paracasei* AR187	87.05 ± 4.60^b^	41.69 ± 2.45^a^	16 ± 2^c^
*L. plantarum* AR171	89.04 ± 6.08^c^	22.96 ± 3.15^b^	–^d^

*^1^Data are presented as means ± standard deviations (n = 3). Superscript letters indicate significantly differences (P < 0.05). “–”, not detectable.*

As shown in Figure 1, the four strains exhibited different adhesion abilities, even with the same species. AR326 and AR269 showed good adhesion ability, whereas the adhesion abilities of the AR187 and AR171 were barely detectable. Consistent with the other studies (Del Re et al., 2000; Schillinger et al., 2005; Tuo et al., 2013), the auto-aggregating and hydrophobicity results did not strongly reflect the adhering ability well.

When AR326 and AR269 were treated with trypsin, the number of bacterial cells adhering to HT-29 cells decreased by more than 50% (Table 2). After the surface protein extracts were added, the adhesion of AR326 and AR269 treated with trypsin treatment significantly increased. Compared to addition of heterologous surface proteins, the addition of the strains’ own surface proteins had more obvious effects. For example, the adhesion ability of AR326 was 347 bacterial counts/100 cells with its own surface protein extracts added, but only 267 bacterial counts/100 cells with the addition of AR269 surface protein extracts. After trypsin treatment, the addition of surface protein extracts restored the lactobacilli’s adhesion ability, indicating that surface proteins play an important role in bacterial adhesion to intestinal epithelial cells.

**Table 2:** Adhesion abilities of the *Lactobacillus* strains with and without trypsin treatment or added surface protein extracts.

Strains	Treatment conditions	Adhesion ability (bacterial counts/100 HT-29 cells)
AR326	No treatment	411 ± 12^a1^
AR269	No treatment	394 ± 21^ab^
AR187	No treatment	16 ± 2^j^
AR171	No treatment	–^k^
AR326	Trypsin	202 ± 34^ef^
AR269	Trypsin	127 ± 32^h^
AR187	Trypsin	–^k^
AR171	Trypsin	–^k^
AR326	Own surface protein extracts added after trypsin treatment	347 ± 38^c^
AR269	Own surface protein extracts added after trypsin treatment	309 ± 23^cd^
AR269	Surface protein extracts from AR326 added after trypsin treatment	291 ± 20^d^
AR326	Surface protein extracts from AR269 added after trypsin treatment	267 ± 33^de^
AR187	Surface protein extracts from AR326 added	150 ± 10^gh^
AR171	Surface protein extracts from AR326 added	104 ± 12^h^
AR187	Surface protein extracts from AR269 added	255 ± 30^de^
AR171	Surface protein extracts from AR269 added	148 ± 23^gh^
AR187	Surface protein extracts from AR326 added after trypsin treatment	57 ± 4^i^
AR171	Surface protein extracts from AR326 added after trypsin treatment	243 ± 33^e^
AR187	Surface protein extracts from AR269 added after trypsin treatment	366 ± 13^b^
AR171	Surface protein extracts from AR269 added after trypsin treatment	345 ± 26^c^

*^1^Data are presented as means ± standard deviations (n = 3). Superscript letters indicate significant differences (P < 0.05). “–”, not detectable.*

AR187 and AR171 with or without treatment had poor adhesion abilities. The adhesion abilities of AR187 and AR171 were significantly improved when surface protein extracts from AR326 or AR269 were added. The numbers of AR187 and AR171 cells adhering to HT-29 cells significantly increased (*p* < 0.05) to 150 and 104 bacterial counts/100 cells, respectively, after the addition of the surface protein extracts originating from AR326, and to 255 and 148 bacterial counts/100 cells after the addition of surface protein extracts originating from AR269. Compared with the strains that were not treated with trypsin, when AR187 and AR171 were treated with trypsin before addition of the surface protein extracts of AR326 or AR269, their adhesive abilities were significantly improved except when surface protein extracts originating from AR326 were added to AR187. The numbers of AR187 and AR171 cells adhering to HT-29 cells significantly increased to 150 and 104 bacterial counts/100 cells, respectively, after addition of the surface protein extracts originating from AR326. The numbers of AR187 and AR171 cells adhering to HT-29 cells even increased to 366 and 345 bacterial counts/100 cells, respectively, after addition of surface protein extracts originating from AR269. These results indicate that the addition of surface protein extracts can effectively increase the adhesion of strains with the poor adhesion ability. However, when heat-treated surface protein extracts were added to the AR171 and AR187 with or without trypsin treatment, their adhesion ability did not change, indicating that denatured surface proteins cannot increase the adhesion abilities of bacteria.

### Characterization of the Surface Protein Extracts Isolated From *L. plantarum* AR326

**FIGURE 2:**
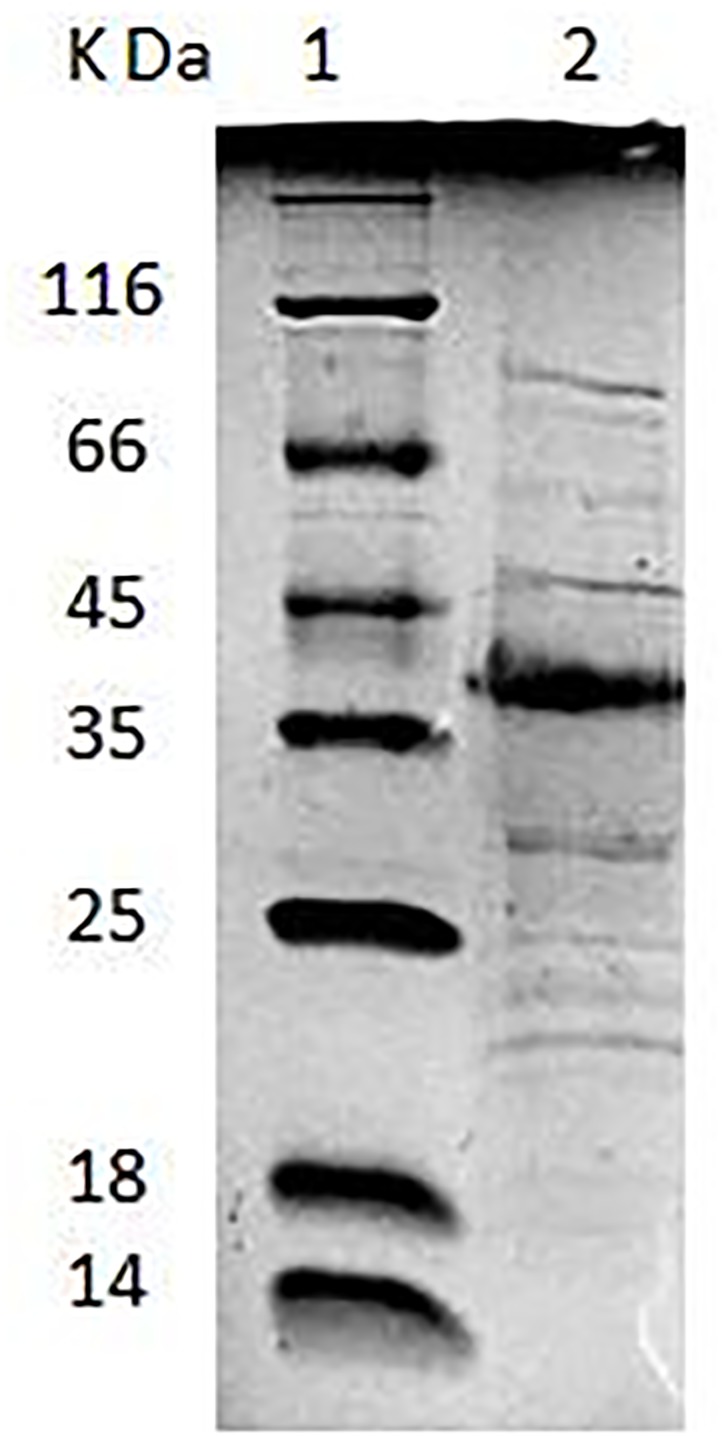
Lane 1: low-molecular-mass protein standard. Lane 2: SDS–PAGE analysis of AR326 surface protein extracted with 5 M LiCl.

*Lactobacillus plantarum* AR326 had the best adhesion ability of the tested strains and its surface protein extracts significantly increased the adhesion ability of strains with the poor adhesion ability. SDS–PAGE was used to identify the major surface protein and mass spectrometry analysis was performed to determine the protein sequence. As shown in Figure 2, an intense band of an approximately 37 kDa was observed, as well as several other less intense bands. The 37 kDa protein was cut out and analyzed by mass spectrometry analysis. The N-terminus sequence of the 37 kDa protein obtained was found to be MSVKIG and showed 100% homology with that of the GAPDH protein of *L. plantarum* WCFS1 (GenBank: YP_004888763) by BLAST search in the NCBI database.

### Preparation of the GAPDH Protein of *L. plantarum* AR326 and Its Antibody

**FIGURE 3:**
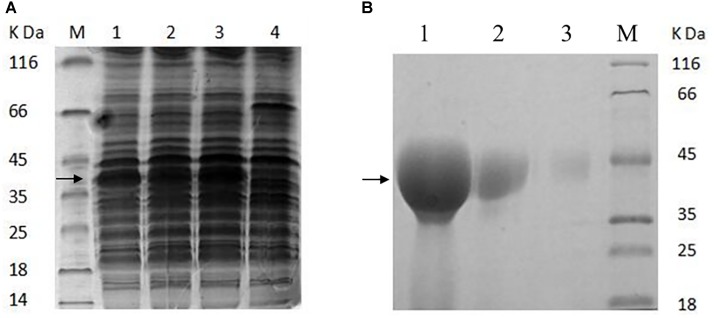
**(A)** SDS–PAGE analysis of the proteins extracted from the recombinant *E. coli* BL21 (DE3) **(A)** and purified GAPDH protein **(B)**. **(A)** Line 1, induced with 0.01 mmol/L of IPTG; line 2, induced with 0.05 mmol/L of IPTG; line 3, induced with 0.1 mmol/L of IPTG; line 4, without inducing; M, low-molecular-mass protein standard. **(B)** Line 1, purified GAPDH protein was concentrated 20 times; line 2, the purified GAPDH protein was concentrated five times; line 3, the purified GAPDH protein (not concentrated); M, low-molecular-mass protein standard.

To confirm the adhesion role of the GAPDH of AR326, the GAPDH protein was expressed in an *E. coli* system and its antibody was prepared. The GAPDH gene was successfully obtained by PCR using the primers GapAF and GapAR, and *L. plantarum* AR326 DNA as the template. The sequence of the PCR product showed 100% homology with that of the GAPDH of *L. plantarum* WCFS1. The constructed plasmid pET24a-GAPDH was transformed into *E. coli* BL21 (DE3) and the expression was induced with different IPTG concentrations (Figure 3A). At all concentrations, GAPDH was successfully expressed in solution. IPTG at 0.1 mmol/L was associated with a high level of the GAPDH protein expression. The expressed protein was subsequently purified by affinity chromatography (Figure 3B). The concentrated GAPDH was used to generate an anti-GAPDH polyclonal antibody in rabbits and used in the subsequent addition experiments. An ELISA test showed the sensitivity of the acquired antibody reached 0.5 μg/mL.

### Anti-GAPDH Antibody Inhibited Adhesion and GAPDH Increased Adhesive Ability

**FIGURE 4:**
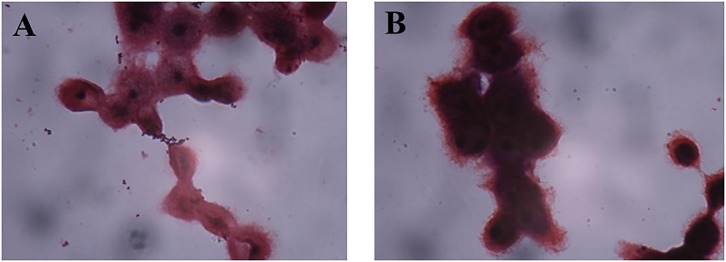
Adhesion of AR269 **(A)** and AR326 **(B)** to HT-29 cells after treatment with anti-GAPDH.

The good adhesive ability of AR326 and AR269 decreased significantly with the addition of anti-GAPDH antibody obtained from AR326 GAPDH (Figure 4 and Table 3). The number of AR326 cells adhering to HT-29 cells significantly decreased (*p* < 0.05) to 20 bacterial counts/100 cells after blocking by an anti-GAPDH rabbit antibody, while the value was 411 bacterial counts/100 cells for the untreated strain, a decrease of approximately 20-fold. In addition, when the anti-GAPDH antibody was heat-treated before addition to the AR326 and AR269, there was no significant difference from the untreated control strain in terms of adhesive ability. When anti-GAPDH antibody and GAPDH were added simultaneously, the adhesive ability of AR326 was significantly higher than that of AR326 with anti-GAPDH antibody treatment but lower than AR326 without any treatment. The adhesive ability of AR269 when anti-GAPDH antibody and GAPDH were simultaneously added was significantly higher than with anti-GAPDH antibody treatment alone, but not significantly different from AR269 without any treatment. These results show that anti-GAPDH antibody can effectively inhibit the adhesion of the strains with higher adhesive ability, especially for AR326. When GAPDH was added to the AR326 and AR269, which had first been treated with trypsin, their adhesion was increased significantly (*p* < 0.05) compared with the strains treated with trypsin alone, and was close to the adhesion of untreated strains (Table 4). These results indicated that GAPDH plays an important role in bacterial adhesion.

**Table 3:** Adhesion abilities of *Lactobacillus* strains treated with anti-GAPDH antibody.

Strains	Treatment conditions	Adhesion ability (bacterial counts/100 HT-29 cells)
AR326	No treatment	411 ± 12^a1^
AR269	No treatment	394 ± 21^ab^
AR187	No treatment	16 ± 2^f^
AR171	No treatment	–^g^
AR326	Added anti-GAPDH antibody	19.65 ± 5.14^f^
AR269	Added anti-GAPDH antibody	225.65 ± 13.69^c^
AR326	Simultaneously added anti-GAPDH antibody and GAPDH	153.91 ± 10.59^d^
AR269	Simultaneously added anti-GAPDH antibody and GAPDH	393.67 ± 5.34^ab^
AR326	Heat-treated anti-GAPDH antibody added	401.23 ± 22.98^a^
AR269	Heat-treated anti-GAPDH antibody added	375.15 ± 19.16^b^

*^1^Data are presented as means ± standard deviations (n = 3). Superscript letters indicate significant differences (P < 0.05). “–”, not detectable.*

**FIGURE 5:**
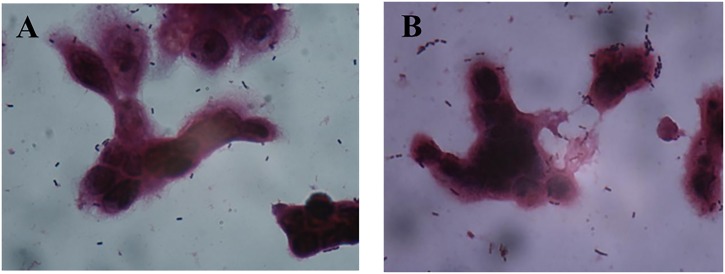
Adhesion of AR171 **(A)** and AR187 **(B)** on to HT-29 cells after treatment with GAPDH as observed by Gram staining under a light microscope (magnification 1000×).

AR171 and AR187 had poor adhesion ability. As shown in Figure 5, treating AR171 and AR187 with purified GAPDH protein resulted in increased binding to HT-29 cells, compared with the untreated strains. After addition of GAPDH, the number of AR171 and AR187 cells adhering to HT-29 cells significantly increased (*p* < 0.05) to 181 and 127 bacterial counts/100 cells, respectively; while the value was close to 0 for the untreated strains (Table 4). In addition, when the four strains were treated with trypsin before the addition of GAPDH, their adhesion abilities were significantly increased compared with the strains treated with trypsin alone (Table 4). For AR171, the adhesion with added GAPDH directly (181 bacterial counts/100 cells) was higher than when the bacteria were treated with trypsin before GAPDH was added (141 bacterial counts/100 cells), but there was no significant difference in the results of these two treatments for AR187. These results indicate that added GAPDH can significantly increase the adhesion of strains with poor adhesion ability.

**Table 4:** Adhesion abilities of *Lactobacillus* strains with added GAPDH.

Strains	Treatment conditions	Adhesion ability (bacterial counts/100 cells)
AR326	No treatment	411 ± 12^a1^
AR269	No treatment	394 ± 21^ab^
AR187	No treatment	16 ± 2^h^
AR171	No treatment	–^i^
AR171	Added purified GAPDH	181.49 ± 20.5^e^
AR187	Added purified GAPDH	127.48 ± 9.26^g^
AR171	GAPDH added after treatment with trypsin	141.91 ± 18.3^fg^
AR187	GAPDH added after treatment with trypsin	93.27 ± 10.92^g^
AR269	GAPDH added after treatment with trypsin	295.19 ± 8.75^d^
AR326	GAPDH added after treatment with trypsin	355.97 ± 16.06^bc^

*^1^Data are presented as means ± standard deviations (n = 3). Superscript letters indicate significant differences (P < 0.05). “–”, not detectable.*

## Discussion

The adhesion abilities of lactobacilli have been linked to their surface properties. A number of surface components are involved in adherence to the epithelial cells, including (lipo) teichoic acids, polysaccharides, and proteins. In this study, the adhesion abilities of *Lactobacillus* strains were significantly reduced with the trypsin treatment, in agreement with previous studies (Deepika and Charalampopoulos, 2010; Jensen et al., 2014), indicating that the surface proteins play an important role in bacterial adhesion. However, the adhesion levels of *L. plantarum* AR326 and AR269 with a high adhesion ability did not drop to the zero, these once demonstrated besides the surface proteins, non-surface proteins, and non-proteinaceous compounds were associated with the adhesion of lactobacilli (Deepika and Charalampopoulos, 2010; Polak-Berecka et al., 2014; Pajarillo et al., 2017; Yadav et al., 2017). Directly adding the surface proteins to the strains with poor adhesion ability effectively increased their adhesion, suggesting that the surface proteins can self-assemble to form an active structure around the bacteria. The denatured surface proteins did not change the adhesion abilities of the strains, indicating that protein structure is important for the adhesion to intestinal cells. Trypsin treatment before the addition of surface proteins increased adhesion ability compared with no trypsin treatment, possibly because the added surface proteins prefer adhering to a “naked” bacterial surface. It has been reported that the bacterial cells treated with trypsin exhibited a significant decrease in hydrophobicity (Deepika et al., 2009). However, *L. paracasei* AR187 and *L. plantarum* AR171, which have similar surface hydrophobicity, had different adhesion abilities after the addition of surface proteins. Surface hydrophobicity may not be the primary determinant for added surface proteins adhering to bacteria surface. Further experiments are required to determine the surface proteins adhesion factors.

As the surface proteins were shown to play a role in bacterial adhesion, we characterized the surface proteins of *L. plantarum* AR326, which had high adhesion ability. The most abundant 37 kDa protein was identified as GAPDH by mass spectrometry analysis, indicating that the GAPDH protein on the bacterial surface is likely to play a role in adhesion to HT-29 cells.

Glyceraldehyde-3-phosphate dehydrogenase is an important enzyme in the glycolytic pathway responsible for the conversion of glyceraldehyde 3-phosphate to D-glycerate 1,3-bisphosphate in the cytoplasm. Recently, many reports have confirmed that GAPDH is also located on the surface of many bacteria, such as *Lactobacillus reuteri* (Zhang W.M. et al., 2015), *Lactobacillus jensenii* (Spurbeck and Arvidson, 2010), and *L. plantarum* (Saad et al., 2009). GAPDH is also recognized as a moonlighting protein. Moonlighting proteins have multiple functions not due to gene fusions, splice variants, or multiple proteolytic fragments (Wang et al., 2014). They appear to be common in bacteria and have been identified in many lactic acid bacteria (Kinoshita et al., 2016). Many previous reports have shown that surface GAPDH can adhere to mucus components, and extracellular matrices, such as fibronectin, laminin, and collagen. A study have found that GAPDH existed on the surface of *Lactobacillus casei* under acidic conditions (Zhang W.M. et al., 2015). These surface components have been implicated in the adhesion role of GAPDH (Glenting et al., 2013; Wang et al., 2014). As GAPDH is a glycolytic enzyme, it is not impossible to study its moonlighting function and potential mechanisms on adhesion by the traditional methods of genetic manipulation, such as a knockout mutant. After the removal of GAPDH or surface protein by guanidine–HCl and LiCl treatment, the adhesive ability of *L. plantarum* 423 and *L. reuteri* ZJ617 was decreased (Carasi et al., 2014; Zhang W.M. et al., 2015). In this study, the high adhesion abilities of *L. plantarum* AR326 and AR269 significantly decreased after blocking with anti-GAPDH antibody. Adding GAPDH restored the adhesion abilities of AR326 and AR269 treated with trypsin. These results directly demonstrate that GAPDH plays an important role in bacteria adhesion.

A main objective of identifying the adhesive proteins is to use them to improve the adhesive ability of the strains with poor adhesive ability. Overexpressing these proteins is the most common method of conferring their adhesive properties with varying degrees. For examples, *Lactococcus lactis* MG1363 expressing cell surface *Streptococcus gordonii* SspB exhibited fivefold-increased binding (Holmes et al., 1998), and CwaA from *L. plantarum* can increase the number of *L. lactis* NZ9000 adhering to HT-29 cells by about 40-fold (Zhang B. et al., 2015). However, these adhesion abilities were decided by expression host and overexpressed adhesive proteins, and their use is restricted because they are engineered strains. GAPDH, which is present in high and constant levels in cells, is often used as a protein normalizing control, making it unsuitable for genetic manipulation such as overexpression. Here, adding GAPDH significantly increased the adhesion of *L. paracasei* AR187 and *L. plantarum* AR171, which have poor adhesive ability. The addition of GAPDH was more effective in strains not treated with trypsin than in those first treated with trypsin. To our knowledge, this is first study demonstrating that directly adding GAPDH can increase the adhesion of strains with poor adhesion ability. These results suggest that GAPDH can be used as supplement directly add to the strains to increase their adhesion ability. The results for AR187 and AR171, which are different species, suggest that GAPDH may be used in a range of species. The mechanism by which GAPDH exercises its adherence function should be investigated in the future studies.

## Conclusion

In this study, we assessed the adhesion abilities of *L. plantarum* AR326, AR269, and AR171 and *L. paracasei* AR187. The good adhesion abilities of *L. plantarum* AR326 and AR269 were significantly decreased after trypsin treatment, and surface proteins extracts can increase the adhesion of AR171 and AR187 with poor adhesion ability and the strains treated with trypsin. By SDS–PAGE and mass spectrometry analysis, we found that the main component of the surface proteins was GAPDH. The anti-GAPDH antibody blocked the adhesion of AR326 and AR269, and GAPDH restored the adhesion of AR326 and AR269 treated with trypsin. In addition, directly added GAPDH significantly increased the adhesion of the strains with the poor adhesion abilities. We therefore expect that GAPDH can be used as supplement for future to increase the adhesion of strains of interest.

## Author Contributions

GW interpreted the results, collected the test data, and drafted the manuscript. MZ and JZ assisted to complete the experiments. JZ assisted to revise the manuscript. YX and LA designed the study. PL polished the manuscript.

## Conflict of Interest Statement

The authors declare that the research was conducted in the absence of any commercial or financial relationships that could be construed as a potential conflict of interest.

## References

[B1] Avall-Jaaskelainen S. Lindholm A. Palva A. (2003). Surface display of the receptor-binding region of the *Lactobacillus brevis* S-layer protein in *Lactococcus lactis* provides nonadhesive lactococci with the ability to adhere to intestinal epithelial cells. *Appl. Environ. Microbiol.* 69 2230–2236. 10.1128/aem.69.4.2230-2236.2003 12676705 PMC154836

[B2] Buck B. L. Altermann E. Svingerud T. Klaenhammer T. R. (2005). Functional analysis of putative adhesion factors in *Lactobacillus acidophilus* NCFM. *Appl. Environ. Microbiol.* 71 8344–8351. 10.1128/AEM.71.12.8344-8351.2005 16332821 PMC1317474

[B3] Carasi P. Ambrosis N. M. De Antoni G. L. Bressollier P. Urdaci M. C. Serradell Mde L. (2014). Adhesion properties of potentially probiotic *Lactobacillus kefiri* to gastrointestinal mucus. *J. Dairy Res.* 81 16–23. 10.1017/S0022029913000526 24168928

[B4] Choi S. B. Lew L. C. Yeo S. K. Nair Parvathy S. Liong M. T. (2015). Probiotics and the BSH-related cholesterol lowering mechanism: a Jekyll and Hyde scenario. *Crit. Rev. Biotechnol.* 35 392–401. 10.3109/07388551.2014.889077 24575869

[B5] Collado M. C. Meriluoto J. Salminen S. (2008). Adhesion and aggregation properties of probiotic and pathogen strains. *Eur. Food Res. Technol.* 226 1065–1073. 10.1007/s00217-007-0632-x

[B6] Deepika G. Charalampopoulos D. (2010). “Chapter 4 - Surface and adhesion properties of Lactobacilli,” in *Advances in Applied Microbiology*, eds Laskin A. Sariaslani S. Gadd G. (Cambridge, MA: Academic Press), 127–152. 10.1016/s0065-2164(10)70004-6 20359456

[B7] Deepika G. Green R. J. Frazier R. A. Charalampopoulos D. (2009). Effect of growth time on the surface and adhesion properties of *Lactobacillus rhamnosus* GG. *J. Appl. Microbiol.* 107 1230–1240. 10.1111/j.1365-2672.2009.04306.x 19486400

[B8] Del Re B. Sgorbati B. Miglioli M. Palenzona D. (2000). Adhesion, autoaggregation and hydrophobicity of 13 strains of *Bifidobacterium longum*. *Lett. Appl. Microbiol.* 31 438–442. 10.1046/j.1365-2672.2000.00845.x 11123552

[B9] Dhanani A. S. Bagchi T. (2013). The expression of adhesin EF-Tu in response to mucin and its role in *Lactobacillus* adhesion and competitive inhibition of enteropathogens to mucin. *J. Appl. Microbiol.* 115 546–554. 10.1111/jam.12249 23663754

[B10] Dimitrijevic R. Ivanovic N. Mathiesen G. Petrusic V. Zivkovic I. Djordjevic B. (2014). Effects of *Lactobacillus rhamnosus* LA68 on the immune system of C57BL/6 mice upon oral administration. *J. Dairy Res.* 81 202–207. 10.1017/S0022029914000028 24559976

[B11] Glenting J. Beck H. C. Vrang A. Riemann H. Ravn P. Hansen A. M. (2013). Anchorless surface associated glycolytic enzymes from *Lactobacillus plantarum* 299v bind to epithelial cells and extracellular matrix proteins. *Microbiol. Res.* 168 245–253. 10.1016/j.micres.2013.01.003 23395591

[B12] Holmes A. R. Gilbert C. Wells J. M. Jenkinson H. F. (1998). Binding properties of Streptococcus gordonii SspA and SspB (antigen I/II family) polypeptides expressed on the cell surface of *Lactococcus lactis* MG1363. *Infect. Immun.* 66 4633–4639. 9746559 10.1128/iai.66.10.4633-4639.1998 PMC108570

[B13] Jensen H. Roos S. Jonsson H. Rud I. Grimmer S. Van Pijkeren J. P. (2014). Role of *Lactobacillus reuteri* cell and mucus-binding protein A (CmbA) in adhesion to intestinal epithelial cells and mucus in vitro. *Microbiology* 160(Pt 4), 671–681. 10.1099/mic.0.073551-0 24473252 PMC7336543

[B14] Kemgang T. S. Kapila S. Shanmugam V. P. Kapila R. (2014). Cross-talk between probiotic lactobacilli and host immune system. *J. Appl. Microbiol.* 117 303–319. 10.1111/jam.12521 24738909

[B15] Kim K. Lee G. Thanh H. D. Kim J. H. Konkit M. Yoon S. (2018). Exopolysaccharide from *Lactobacillus plantarum* LRCC5310 offers protection against rotavirus-induced diarrhea and regulates inflammatory response. *J. Dairy Sci.* 101 5702–5712. 10.3168/jds.2017-14151 29627242

[B16] Kim Y. W. Jeong Y. J. Kim A. Y. Son H. H. Lee J. A. Jung C. H. (2014). *Lactobacillus brevis* strains from fermented aloe vera survive gastroduodenal environment and suppress common food borne enteropathogens. *PLoS One* 9:e90866. 10.1371/journal.pone.0090866 24598940 PMC3944883

[B17] Kinoshita H. Ohuchi S. Arakawa K. Watanabe M. Kitazawa H. Saito T. (2016). Isolation of lactic acid bacteria bound to the porcine intestinal mucosa and an analysis of their moonlighting adhesins. *Biosci. Microbiota Food Health* 35 185–196. 10.12938/bmfh.16-012 27867805 PMC5107636

[B18] Kobyliak N. Conte C. Cammarota G. Haley A. P. Styriak I. Gaspar L. (2016). Probiotics in prevention and treatment of obesity: a critical view. *Nutr. Metab.* 13:14. 10.1186/s12986-016-0067-0 26900391 PMC4761174

[B19] Ljungh A. Wadstrom T. (2006). Lactic acid bacteria as probiotics. *Curr. Issues Intest. Microbiol.* 7 73–89. 16875422

[B20] Lortal S. Van Heijenoort J. Gruber K. Sleytr U. B. (1992). S-layer of *Lactobacillus helveticus* ATCC 12046: isolation, chemical characterization and re-formation after extraction with lithium chloride. *Microbiology* 138 611–618. 10.1099/00221287-138-3-611

[B21] Marteau P. Seksik P. Jian R. (2002). Probiotics and intestinal health effects: a clinical perspective. *Br. J. Nutr.* 88(Suppl. 1), S51–S57. 10.1079/BJN2002629 12215185

[B22] Miyoshi Y. Okada S. Uchimura T. Satoh E. (2006). A mucus adhesion promoting protein, MapA, mediates the adhesion of *Lactobacillus reuteri* to Caco-2 human intestinal epithelial cells. *Biosci. Biotechnol. Biochem.* 70 1622–1628. 10.1271/bbb.50688 16861796

[B23] Mustapha A. Jiang T. Savaiano D. A. (1997). Improvement of lactose digestion by humans following ingestion of unfermented acidophilus milk: influence of bile sensitivity, lactose transport, and acid tolerance of *Lactobacillus acidophilus*. *J. Dairy Sci.* 80 1537–1545. 10.3168/jds.S0022-0302(97)76083-1 9276791

[B24] Oak S. J. Jha R. (2018). The effects of probiotics in lactose intolerance: a systematic review. *Crit. Rev. Food Sci. Nutr.* 10.1080/10408398.2018.1425977 [Epub ahead of print]. 29425071

[B25] Olson B. J. Markwell J. (2007). Assays for determination of protein concentration. *Curr. Protoc. Protein Sci.* 48 3.4.1–3.4.29. 10.1002/0471140864.ps0304s48 18429326

[B26] Pajarillo E. A. B. Kim S. H. Valeriano V. D. Lee J. Y. Kang D. K. (2017). Proteomic view of the crosstalk between *Lactobacillus mucosae* and intestinal epithelial cells in co-culture revealed by Q exactive-based quantitative proteomics. *Front. Microbiol.* 8:2459. 10.3389/fmicb.2017.02459 29312173 PMC5732961

[B27] Polak-Berecka M. Wasko A. Paduch R. Skrzypek T. Sroka-Bartnicka A. (2014). The effect of cell surface components on adhesion ability of *Lactobacillus rhamnosus*. *Antonie Van Leeuwenhoek* 106 751–762. 10.1007/s10482-014-0245-x 25090959 PMC4158178

[B28] Pretzer G. Snel J. Molenaar D. Wiersma A. Bron P. A. Lambert J. (2005). Biodiversity-based identification and functional characterization of the mannose-specific adhesin of *Lactobacillus plantarum*. *J. Bacteriol.* 187 6128–6136. 10.1128/JB.187.17.6128-6136.2005 16109954 PMC1196140

[B29] Saad N. Urdaci M. Vignoles C. Chaignepain S. Tallon R. Schmitter J. M. (2009). *Lactobacillus plantarum* 299v surface-bound GAPDH: a new insight into enzyme cell walls location. *J. Microbiol. Biotechnol.* 19 1635–1643. 10.4014/jmb.0902.0102 20075631

[B30] Schillinger U. Guigas C. Holzapfel W. H. (2005). In vitro adherence and other properties of *Lactobacilli* used in probiotic yoghurt-like products. *Int. Dairy J.* 15 1289–1297. 10.1016/j.idairyj.2004.12.008

[B31] Sinclair A. Xie X. Saab L. Dendukuri N. (2016). *Lactobacillus probiotics* in the prevention of diarrhea associated with *Clostridium difficile*: a systematic review and Bayesian hierarchical meta-analysis. *CMAJ Open* 4 E706–E718. 10.9778/cmajo.20160087 28018885 PMC5173486

[B32] Spurbeck R. R. Arvidson C. G. (2010). *Lactobacillus jensenii* surface-associated proteins inhibit *Neisseria gonorrhoeae* adherence to epithelial cells. *Infect. Immun.* 78 3103–3111. 10.1128/IAI.01200-09 20385752 PMC2897381

[B33] Staudacher H. (2015). Probiotics for lactose intolerance and irritable bowel syndrome. *Br. J. Commun. Nurs. Suppl. Nutr.* 20(Suppl. 6a), S12–S14. 10.12968/bjcn.2015.20.Sup6a.S12 26087202

[B34] Tuo Y. Song X. Song Y. Liu W. Tang Y. Gao Y. (2018). Screening probiotics from *Lactobacillus* strains according to their abilities to inhibit pathogen adhesion and induction of pro-inflammatory cytokine IL-8. *J. Dairy Sci.* 101 4822–4829. 10.3168/jds.2017-13654 29550135

[B35] Tuo Y. Yu H. Ai L. Wu Z. Guo B. Chen W. (2013). Aggregation and adhesion properties of 22 *Lactobacillus* strains. *J. Dairy Sci.* 96 4252–4257. 10.3168/jds.2013-6547 23664349

[B36] Ventura M. Jankovic I. Walker D. C. Pridmore R. D. Zink R. (2002). Identification and characterization of novel surface proteins in *Lactobacillus johnsonii* and *Lactobacillus gasseri*. *Appl. Environ. Microbiol.* 68 6172–6181. 10.1128/aem.68.12.6172-6181.2002 12450842 PMC134427

[B37] Vlková E. Rada V. Šmehilová M. Killer J. (2008). Auto-aggregation and Co-aggregation ability in *Bifidobacteria* and *Clostridia*. *Folia Microbiol.* 53 263–269. 10.1007/s12223-008-0040-z 18661306

[B38] Von Ossowski I. Satokari R. Reunanen J. Lebeer S. De Keersmaecker S. C. Vanderleyden J. (2011). Functional characterization of a mucus-specific LPXTG surface adhesin from probiotic *Lactobacillus rhamnosus* GG. *Appl. Environ. Microbiol.* 77 4465–4472. 10.1128/AEM.02497-10 21602388 PMC3127685

[B39] Wang G. Xia Y. Cui J. Gu Z. Song Y. Chen Y. Q. (2014). The roles of moonlighting proteins in bacteria. *Curr. Issues Mol. Biol.* 16 15–22. 10.21775/cimb.016.015 23872606

[B40] Xie Q. Pan M. Huang R. Tian X. Tao X. Shah N. P. (2016). Short communication: modulation of the small intestinal microbial community composition over short-term or long-term administration with *Lactobacillus plantarum* ZDY2013. *J. Dairy Sci.* 99 6913–6921. 10.3168/jds.2016-11141 27320669

[B41] Yadav A. K. Tyagi A. Kumar A. Panwar S. Grover S. Saklani A. C. (2017). Adhesion of *Lactobacilli* and their anti-infectivity potential. *Crit. Rev. Food Sci. Nutr.* 57 2042–2056. 10.1080/10408398.2014.918533 25879917

[B42] Zhang B. Zuo F. Yu R. Zeng Z. Ma H. Chen S. (2015). Comparative genome-based identification of a cell wall-anchored protein from *Lactobacillus plantarum* increases adhesion of *Lactococcus lactis* to human epithelial cells. *Sci. Rep.* 5:14109. 10.1038/srep14109 26370773 PMC4572922

[B43] Zhang W. M. Wang H. F. Gao K. Wang C. Liu L. Liu J. X. (2015). *Lactobacillus reuteri* glyceraldehyde-3-phosphate dehydrogenase functions in adhesion to intestinal epithelial cells. *Can. J. Microbiol.* 61 373–380. 10.1139/cjm-2014-0734 25867279

[B44] Zhang M. Wang G. Xia Y. Zhang H. Xiong Z. Jia G. (2017). Relationships between adhesion abilities and surface properties of *Lactobacillus plantarum*. *Ind. Microbiol.* 47 37–42.

[B45] Zhang W. Wang H. Liu J. Zhao Y. Gao K. Zhang J. (2013). Adhesive ability means inhibition activities for *Lactobacillus* against pathogens and S-layer protein plays an important role in adhesion. *Anaerobe* 22 97–103. 10.1016/j.anaerobe.2013.06.005 23792230

[B46] Zhu D. Sun Y. Liu F. Li A. Yang L. Meng X. C. (2016). Identification of surface-associated proteins of *Bifidobacterium* animalis ssp. lactis KLDS 2.0603 by enzymatic shaving. *J. Dairy Sci.* 99 5155–5172. 10.3168/jds.2015-10581 27132091

